# Availability of MudPIT data for classification of biological samples

**DOI:** 10.1186/2043-9113-3-1

**Published:** 2013-01-14

**Authors:** Dario Di Silvestre, Italo Zoppis, Francesca Brambilla, Valeria Bellettato, Giancarlo Mauri, Pierluigi Mauri

**Affiliations:** 1, Institute for Biomedical Technologies (ITB-CNR), via F.lli Cervi 93, Segrate (Milan), Italy; 2 Department of Informatics, Systems and Communication, Viale Sarca 336, University of Milano-Bicocca, Milan, Italy

**Keywords:** Sample classification, MudPIT, SVM, Clinical proteomics, Label-free quantification

## Abstract

**Background:**

Mass spectrometry is an important analytical tool for clinical proteomics. Primarily employed for biomarker discovery, it is increasingly used for developing methods which may help to provide unambiguous diagnosis of biological samples. In this context, we investigated the classification of phenotypes by applying support vector machine (SVM) on experimental data obtained by MudPIT approach. In particular, we compared the performance capabilities of SVM by using two independent collection of complex samples and different data-types, such as mass spectra (*m/z*), peptides and proteins.

**Results:**

Globally, protein and peptide data allowed a better discriminant informative content than experimental mass spectra (overall *accuracy* higher than 87% in both collection 1 and 2). These results indicate that sequencing of peptides and proteins reduces the experimental noise affecting the raw mass spectra, and allows the extraction of more informative *features* available for the effective classification of samples. In addition, proteins and peptides *features* selected by SVM matched for 80% with the differentially expressed proteins identified by the MAProMa software.

**Conclusions:**

These findings confirm the availability of the most label-free quantitative methods based on processing of spectral count and SEQUEST-based SCORE values. On the other hand, it stresses the usefulness of MudPIT data for a correct grouping of sample phenotypes, by applying both supervised and unsupervised learning algorithms. This capacity permit the evaluation of actual samples and it is a good starting point to translate proteomic methodology to clinical application.

## Background

The identification of proteins changing their quantitative level is a key aspect to investigate biological systems as well as to develop strategies for classifying samples into pre-specified categories, such as healthy and diseased. In fact, one of the main objectives of the clinical proteomics is to use relevant biomarkers for improving disease diagnosis or for monitoring the efficacy of treatments [1].

A procedure for discriminating biological samples involves a preliminary evaluation of experimental data, useful for building classification models [2]. In this context, a wide variety of algorithms has been used for processing raw mass spectra, mainly generated by MALDI [3-10] and SELDI technologies [11-14]. Although results from diagnostic studies based on SELDI have generated both excitement and scepticism, it doesn’t allows a direct identification of proteins and it is based on *m/z* signals, only. On the other hand, MALDI is mainly used for the identification of peptides and its reproducibility is strongly dependent by sample preparation method. Besides, in many studies, selected discriminant mass spectrometry signals have then been identified by liquid chromatography (LC) coupled to mass spectrometry (MS). Nevertheless, few works have directly taken into consideration LC-MS data for discriminating biological samples [15,16]. On the contrary, some authors have used them, combined to machine learning algorithms, for improving tandem mass (MS/MS) spectra quality assessment and hence, the protein identification [17-20].

Recently, the improvement of robustness and reproducibility of the MudPIT (Multidimensional Protein Identification Technology) approach, based on two dimensional liquid chromatography coupled to tandem mass spectrometry, has permitted a correct grouping of phenotypes, by using unsupervised algorithms [21-23]. Based on these findings, MudPIT may represent an attractive methodology for improving methods concerning sample classification. It allows to automatically obtain thousands of *features* comprising spectra, peptide sequences and related proteins [24,25]. In addition, label-free quantification approaches based on spectral count (SpC) or SEQUEST-based SCORE evaluation permit an high-throughput discovering of multiple biomarkers [26-28], which could contain a higher level of discriminatory information.

The present study investigates in-depth the availability of MudPIT data for the classification of biological samples. We focused on classification performances achievable by processing different data-types, such as spectra, peptides and proteins. Specifically, we applied a class of machine learning algorithms, i.e. Support Vector Machine (SVM), to identify most predictive *features* and to score the data-types according to the inference performances of the algorithm [29,30]. Finally, since the identification of *features* allowing a model of classification is a key challenge for high-dimensional data, we evaluated how the applied selection method correlates with an independent label-free quantification approach. Therefore, we measured the overlapping of the *features* selected by SVM with the differentially expressed proteins selected by means of the MAProMa software [31].

## Methods

### Data collections

For the study purpose, two pre-existing different collections of experimental data were used. They were previously obtained by means of MudPIT analysis of complex samples, such as the adipose and cardiac tissues, collected according to the acquisition of the informed consent of patients, and the ethical approval of protocols for the care and use of laboratory animals, as reported by Brambilla *et al.*[32] and Simioniuc *et al.*[23], respectively. Specifically, for collection 1 were considered 30 diseased and 11 healthy controls, while 18 diseased and 18 healthy controls were considered for collection 2 (Additional file 1: Figure S1). Experimental details of the MudPIT analysis are reported in Additional file 2.

### Data handling of MS results

Raw mass spectra (MS) produced by MudPIT were handled using MZmine software [33]. Peak detection was performed by the chromatogram builder module by using the Centroid algorithm. Each file containing MS spectra was processed individually and converted to pairs of *m/z* and intensity values by considering all data points above the specified noise level (*e*^3^). Then, *m/z* data points were connected to form chromatograms. In particular, the minimum time span was set to 1 min, the minimum absolute height to *e*^3^and the *m/z* tolerance to 0.5. Finally, peak lists were aligned by Join aligner method applying a ranges of tolerance of 0.5 and 1 min for mass and retention time, respectively.

The experimental tandem mass spectra (MS/MS) were correlated to *in-silico* tryptic peptide sequences, and accordingly to parent proteins, by using a database search method based on the SEQUEST algorithm [34]. The validity of spectrum/peptide matching was assessed using SEQUEST defined parameter thresholds (Additional file 2). Finally, protein and peptide lists obtained from each sample were handled and aligned using MAProMa software and an in-house R-script, respectively [31,35].

In order to evaluate the reproducibility of the MudPIT approach, protein lists of technical replicates were aligned and then processed using a linear-regression-based analysis:

(1) $$Y_{i}=\beta_{0}+\beta_{1}X_{i}+u_{i}$$

where: *i* = 1,*..*,*n*; with *n* = number of variables (proteins) *Y*_*i*_ is the spectral count (SpC) value of the protein *i* in the first replicate analysis *X*_*i*_ is the spectral count (SpC)) value of the protein *i* in the second replicate analysis *β*_0_ is the intercept of the regression line of the population *β*_1_ is the slope or gradient of the regression line of the population *u*_*i*_ is the error term

### Proteomic datasets

Each sample belonging to the collection 1 and 2 was represented by five different datasets, including the global protein/peptide profiles and *m/z* precursor ions from three different chromatographic steps (60, 120, 400 mM) of the applied analytical method (Additional file 2). Each dataset was formatted in a *s*×*f* matrix, where *s* represents the number of samples and *f * the number of *features*. Entries of the protein data matrix were the spectral count (SpC) values assigned by the SEQUEST algorithm to each identified protein; in the same way, Xcorrelation values and peak area intensity (AUC) were used for the peptide and mass spectra data matrices, respectively (Additional file 3: Table S1).

### Label-free quantification approach

Proteins differentially expressed between the considered phenotype groups were identified by using a label-free quantification approach. In particular, SEQUEST-based SCORE values were processed by means of the DAve and DCI formulas, which are inserted in MAProMa software [31]. In addition, SpC values were evaluated by using the G-test [36] and the unpaired Student’s t-test. In this scenario, proteins with DAve ≥ 0.3 (≤ −0.3) and DCI ≥ 300 (≤ −300), or statistical meaningful at least for one test (*P* > 95 *%*) were considered for the study purpose (Additional file 2 and Additional file 4: Figure S2).

### Evaluation procedures by SVM

In order to investigate on the classification performance achievable by the different data-types (spectra, peptides and proteins) we designed specific *Rapid Miner* workflows (RM-WF) mainly addressed to implement a class of algorithms widely used in the *machine learning* community, i.e., the Support Vector Machine (SVM) [30].

In our investigation we sequentially applied two main operational processes i.e., feature selection and model construction (and validation), respectively. We briefly summarize in the following issues the RM-WF designed for each phase (a complete description of each operator is reported in Additional file 5: Figure S3). 

1. **Feature selection phase.** Due to the high number of signals, *features selection* may be helpful to improve both the inference quality and the data understanding. For this reason we first applied a standard feature selection procedure [29]. Broadly speaking we weighted each signal by an information theory criterion (i.e., *info–gain ratio*[37]). Then we considered to employ in the forward phase only signals having a weight greater than 0.6; this way, only 10 signals were considered. The RM-WF in this case is simple, providing only the *info–gain* weighting capability as reported in Additional file 5: Figure S3 (a).

2. **Model construction and validation phase.** To evaluate the classification performance achievable by the different data-types, we employed SVM algorithms as “black boxes” to score each input data-type, according to the inference performances of the algorithm [29]. In order to avoid *over-fitting* we first sub-sampled a set of different data instances: i.e., for each data set, this phase was applied on (data) instances never used in the above feature selection step. Then, for each instance, we considered only intensity (and counting) values corresponding to the previously suggested 10 signals (i.e., feature selection). This approach has been applied together with an optimization procedure to learn the algorithm parameters. As a matter of fact, different learning model may have many parameters, and often it is not clear which values are best for the learning task at hand; in our case, SVMs involve different *kernel* types and, in turn, each of such functions uses specific values which we need to define in the learning algorithms [30]. In order for the SVMs to perform as better (and homogeneous) as possible for each data-type, we optimized such parameters over the same space of common values. That is, we searched the best parameter values (i.e., providing the highest SVM inference performances) among all the combinations of common ranges for each input data collection. The RM-WF reported in Additional file 5: Figure S3 (b) specifies the main steps used in this phase.

Finally, standard indices (*i.e.*, sensitivity, specificity, positive (PPV), negative predictive (NPV), accuracy, F-score, balanced accuracy, informedness and Matthews correlation coefficient) were used as performance measures to verify which data-types provide the best SVM classification [2].

## Results and discussion

**Figure 1:**
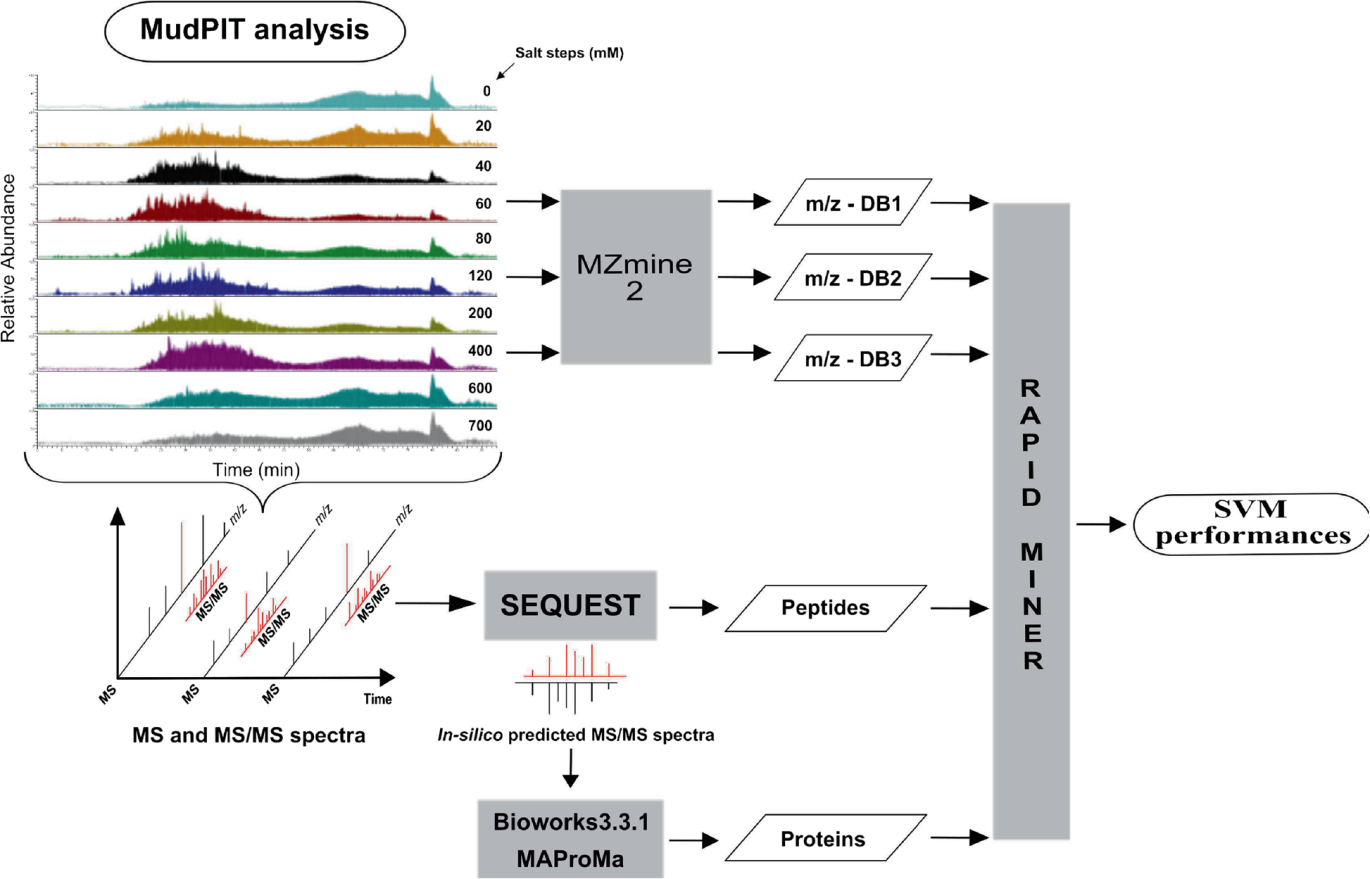
**MudPIT workflow.** Enzymatic digested peptides are first separated by Strong Cation Exchange (SCX), using steps of increasing salt concentration, followed by Reverse Phase (RP) chromatography, using an acetonitrile gradient. Eluted peptides are then directly analyzed by tandem mass spectrometry producing MS and MS/MS spectra. By specific algorithm, such as SEQUEST, and applying appropriate criteria of data filtering (see Additional file 2), the comparison of experimental MS and MS/MS spectra with those *in-silico* predicted from a protein sequence database allows the characterization of the peptide sequences and the corresponding proteins, without limits of isoelectric point (pI), molecular weight (Mw) or hydrophobicity. Using MudPIT, five different datasets per sample were collected for the study purposes. Specifically, in addition to complete protein and peptide profiles, *m/z* data, corresponding to 60 mM, 120 mM, 400 mM of salt concentration steps, were mined collecting three different datasets of spectra.

In this study, we investigated the classification of phenotypes by applying support vector machine (SVM) algorithms on experimental data obtained by MudPIT approach (Figure 1). Identified proteins, peptides and experimental mass spectra (*m/z*) were processed to evaluate the generalization capability of SVM about the *disease vs. healthy* cases used in this study (Additional file 1: Figure S1). For this purpose, a RapidMiner workflow was implemented (Additional file 4: Figure S3). Firstly, a set of data was used as input to SVM learning algorithm. Some learning parameters were optimized over the same common space of values [30]. Finally, data were evaluated according to the inference performance of the algorithm by using standard indices broadly applied to measure the precision and the recall capability [2]. 

By applying a standard *features* selection procedure, ten *features* having a weight greater than 0.6 were selected from each dataset (see features selection phase in Materials and Methods). Model delivered by the SVM operator was applied on independent validation datasets for estimating the performances concerning the phenotype classification. Tables 1 and 2, reporting the standard indices, show the diagnostic capabilities of SVM by using two independent collection of samples and different data-types. Of note, the results suggest that SVM allows a better classification capability by using proteins and peptides rather than mass spectra datasets. In fact, better values of accuracy, F-score, informedness and MCC were observed by considering both collection 1 and collection 2. As opposite, samples classification by means of *m/z* data, resulted to be more difficult. In particular, by using the mass spectra of the collection 1 low values of specificity were observed, while the mass spectra of the collection 2 allowed low overall classification accuracy values.

**Table 1:** Performance of classification obtained by using SVM - Collection1

	**Spec.**	**Sens.**	**PPV**	**NPV**	**Acc.**	**F-score**	**Bal. Acc.**	**Informedness**	**MCC**
Proteins	75%	91%	75%	91%	87%	0.46	83%	67%	0.66
Peptides	75%	100%	100%	92%	94%	0.48	88%	75%	0.72
*m/z*-DB1	50%	96%	80%	85%	84%	0.45	72%	46%	0.43
*m/z*-DB2	75%	96%	86%	92%	90%	0.47	85%	71%	0.69
*m/z*-DB3	37%	100%	100%	83%	84%	0.45	68%	37%	0.34

Specificity (Spec.), sensitivity (Sens.), positive predictive value (PPV), negative predictive value (NPV), accuracy (Acc.), F-score, balanced accuracy (Bal. Acc.), informedness and Matthews correlation coefficient (MCC) of collection 1. Evaluation capabilities have been obtained using observations not considered in the signal selection phase.

**Table 2:** Performance of classification obtained by using SVM - Collection2

	**Spec.**	**Sens.**	**PPV**	**NPV**	**Acc.**	**F-score**	**Bal. Acc.**	**Informedness**	**MCC**
Proteins	93%	93%	93%	93%	93%	0.46	92%	85%	0.85
Peptides	100%	100%	100%	100%	100%	0.50	100%	100%	1
*m/z*-DB1	62%	92%	89%	71%	77%	0.40	77%	54%	0.47
*m/z*-DB2	77%	77%	77%	77%	77%	0.38	77%	54%	0.54
*m/z*-DB3	85%	85%	85%	85%	85%	0.42	84%	70%	0.69

Specificity (Spec.), sensitivity (Sens.), positive predictive value (PPV), negative predictive value (NPV), accuracy (Acc.), F-score, balanced accuracy (Bal. Acc.), informedness and Matthews correlation coefficient (MCC) of collection 2. Evaluation capabilities have been obtained using observations not considered in the signal selection phase.

**Figure 2:**
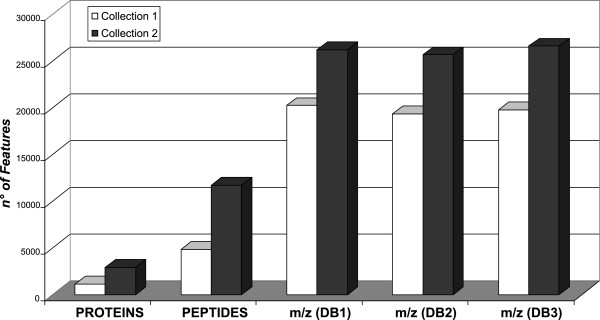
***Features *selected for the study purposes.** Number of *features* (*m/z* ions, peptides and proteins) collected analyzing, by MudPIT, all samples belonging to collection 1 and collection 2. DB1, DB2 and DB3 correspond to *m/z* data mined from 60 mM, 120 mM, 400 mM of salt concentration steps, respectively.

The different classification performances, obtained by SVM, may be related to the *m/z* data complexity. In this regard, an overview of the data was performed by means of Principal Component Analysis (PCA) [38]. As opposed to protein and peptide data, PCA showed that mass spectra, especially for the collection 1, didn’t allow a clear differentiation in the multidimensional space between disease and healthy groups (Additional file 6: Figure S4). In this context, the great amount of mass spectra can make it difficult their data-mining. In fact, a single step of liquid chromatography separation allows the collection of a number of *features* (*m/z* values) about 15 and 3 times bigger than protein and peptide ones, respectively (Figure 2). This great amount of data may be due to the redundant acquisition of spectra, like so to the biological and/or chemical modifications of peptides/proteins (e.g. Post Translational Modifications). Moreover, *m/z* values may be affected by chemical noise as well as to day-to-day instrument variations. Therefore, preprocessing of the raw data significantly influences the quality of the classification results [39,40]. Nevertheless, further errors may be introduced during spectra alignment, while overlapping of *m/z* regions may create ambiguities for peak detection leading to increase the noise and to loss of information and discriminatory ability. 

**Figure 3:**
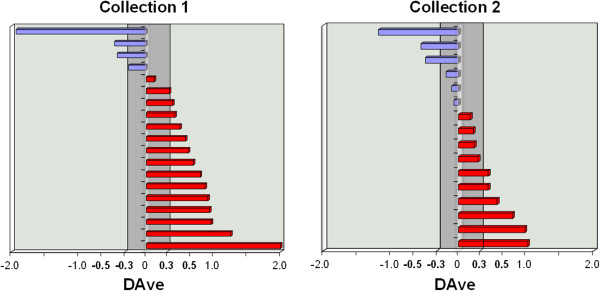
**DAve values for proteins and peptides selected by SVM.** DAve evaluates changes in protein expression and is defined as: ((*X*−*Y*)/(*X* + *Y*))/0.5, while DCI, which describes the confidence of differential expression, is defined as: (*X* + *Y*)∗(*X*−*Y*))/2, where *X* and *Y * represent the SEQUEST-based SCORE values (or spectral count) of a given protein in two compared samples. Conventionally, signs (+/-) of DAve (and DCI) indicate if proteins are up-regulated in the first or in the second sample, respectively.

**Figure 4:**
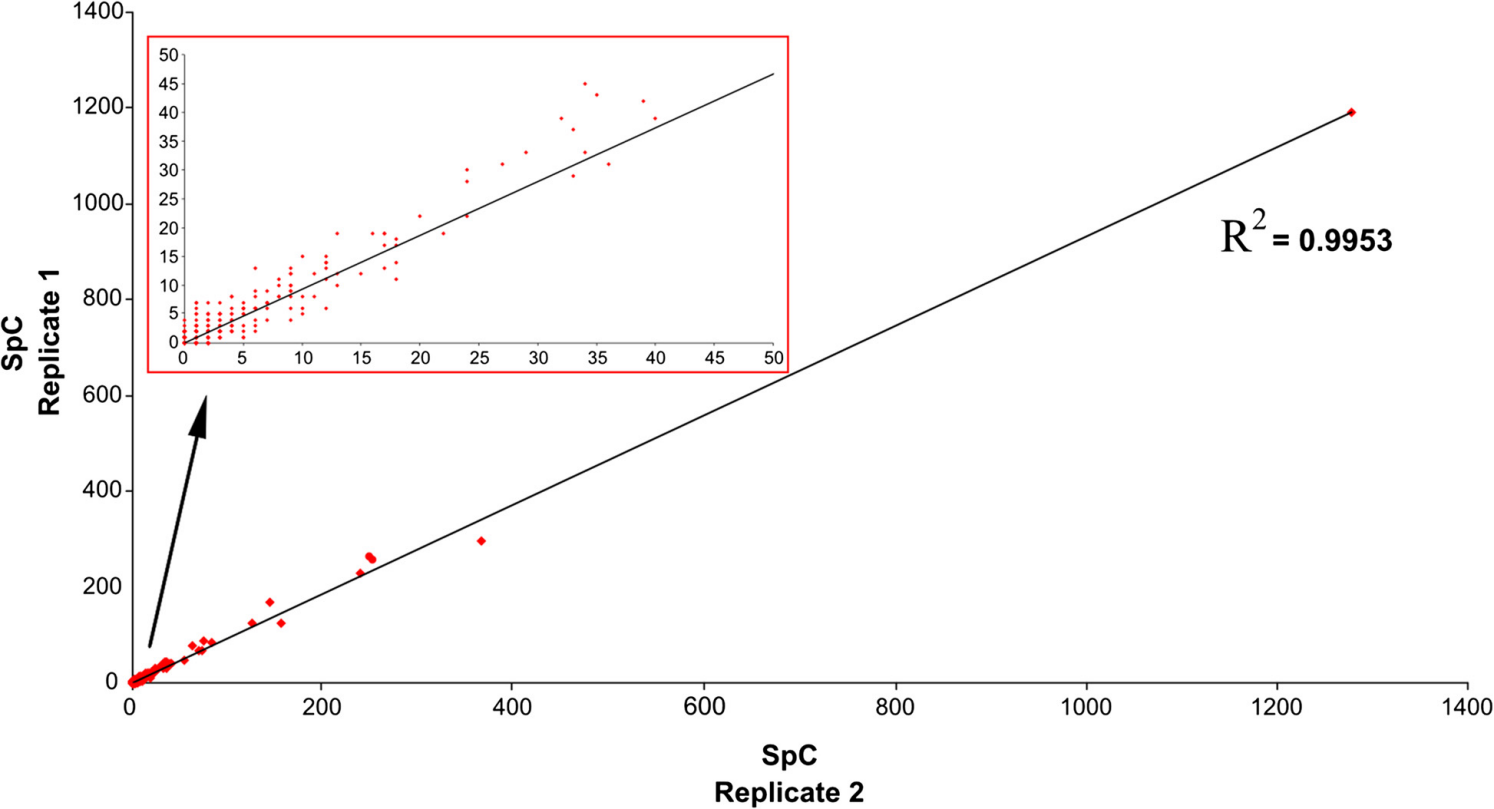
**MudPIT repeatability.** Linear regression analysis obtained by considering SpC values of proteins identified in two technical replicates of MudPIT analysis. R2 and Slope values resulted near to 1. Red rectangle highlights the proteins identified with a low number of peptides and which represent the portion of data less reproducible.

The identification of peptides and proteins by means of the interpretation of tandem mass spectra, can represent a cleaning and a simplifying of *m/z* data complexity. This aspect probably improved the *features* selection process and consequently the performance of classification by means of SVM model. For each collection about 20% of the selected features resulted common between protein and peptide datasets. Besides, around 80% of proteins and peptides, selected by SVM, matched with the differentially expressed proteins selected by MAProMa software (Figure 3). This correspondence represents a mutual validation of these two different procedures and it means that differentially expressed proteins may be used also for a correct grouping of sample phenotypes. For this reason, the use statistical parameters associated with identified proteins and peptides represents a robust procedure for a rapid extraction of potential biomarkers. In addition, MudPIT approach allows a good analytical reproducibility (Figure 4). In fact, although only 60-80 % of protein are identified in two replicate analyses, most of the variation is due to low abundance proteins which are usually identified with a low number of peptides. However, a statistical model has been proposed for estimating the number of replicates required for saturated sampling of a complex protein mixture [41]. 

Our findings are in good agreement with the most widely used semi-quantitative methods concerning the identification of biomarkers using LC-MS/MS approach [25,27]. As for the identification of clinically useful biomarkers, in the last decade, SELDI-TOF analysis has been widely used and many diseases have been mainly studied by serum/plasma protein profiling. Although preliminary results have generated a lot of expectations, later scepticism resulted prevalent [42]. The reasons of this failure is probably due to SELDI profiling based on *m/z* signals, only, and it doesn’t permits a direct identification and quantification of peptides/proteins. In addition, blood samples, although relatively simple to be collected, have a very complex composition with the presence of prominent and unspecific changes, resulting a drawback for the biomarker discovery based on *m/z* signals. On the contrary, we have evidenced in the present manuscript the improved availability of peptide/protein outcomes to allow biomarker discovery and phenotype discrimination. In comparison to mass spectra, sequenced proteins and peptides are less affected by experimental errors, and their use can be useful to avoid the problems of reproducibility due to different instrumental settings occurring over time. In addition, model of healthy/disease tissues represents a source of biomarkers in higher concentration than to plasma, which may be considered mainly useful in their monitoring using other LC-MS procedures [43].

## Conclusion

To realize the potential of MS-based proteomics in the context of clinical utility, for disease diagnosis and prognosis, comparative studies are of great importance. In the present work, MudPIT data, both experimental mass spectra and sequenced peptides/proteins, were processed by SVM for evaluating the corresponding performances of classification. The overall *accuracy* resulted in all investigated cases higher than 77%. In particular, proteins/peptides allowed a better discriminant informative content than experimental mass spectra (overall *accuracy* higher than 87% in both collection 1 and 2). This result is probably due to the translation of mass spectra to peptides/proteins, that eliminates the experimental noise and highlight the actual *features* useful for the phenotype classification. Overall, the presented findings indicate that the impressive amount of data produced by MudPIT approach can be processed for identifying multiple biomarkers and for classifying biological samples, by applying both supervised and unsupervised algorithms. These procedures permit the evaluation of actual samples and translate proteomic methodology to clinical application. In this context, MudPIT approach can be a useful tool for improving the extraction of informative *features* and therefore diagnosis procedures. Probably, in the next future new and more efficient algorithms will be applied, and the discovered biomarkers will be validated by means of fast and high-resolution mass spectrometry and data independent analysis [44,45]. These aspects will be of primary importance to be combined with clinical data and for investigating mechanisms of pathogenesis. In fact, the improved quality of data has the potential to optimize existing protein quantification methods and address the increasing demand of systems biology studies for correlating molecular expression to biological processes.

## Abbreviations

SVM: Support Vector Machine; MudPIT: Multidimensional Protein Identification Technology; MAProMa: Multidimensional Algorithm Protein Map; MALDI: Matrix-Assisted Laser Desorption/Ionization; SELDI: Surface-Enhanced Laser Desorption/Ionization; LC: Liquid Chromatography; MS: Mass Spectrometry; MS/MS: Tandem Mass Spectra; SpC: Spectral Count; DAve: Differential Average; DCI: Differential Confidence Index; RM-WF: Rapid Miner - WorkFlow; PPV: Positive Predicted Value; NPV: Negative Predicted Value; MCC: Matthews Correlation Coefficient; PCA: Principal Component Analysis.

## Competing interests

The authors declare that they have no competing interests.

## Authors’ contributions

DDS and IZ carried out the processing of the experimental data, the interpretation of the results and wrote the manuscript; FB performed the MudPIT experiments; VB carried out the administrative management, GM and PLM conceived the project, participated in the design of the study and in the writing of the manuscript. All authors read and approved the final manuscript.

## Authors’ information

DDS (PhD), Permanent Researcher at the Proteomic and Metabolomic Department of the Institute of Biomedical Technologies - National Research Council (ITB-CNR), located in Segrate (Milan), Italy. His skills include the bioinformatic processing of proteomic data and their functional characterization by using data-derived systems biology approach. IZ (PhD), Permanent Researcher at the Computer Science Department of the University of Milano-Bicocca, located in Milan (Italy). His skills include the machine learning applications to bioinformatics and computational systems biology. FB (PhD), Researcher at the Proteomic and Metabolomic Department of the Institute of Biomedical Technologies - National Research Council (ITB-CNR), located in Segrate (Milan), Italy. His skills include the high-throughput proteomic analysis of complex biological samples by means of the MudPIT methodology. VB, Technician at the Proteomic and Metabolomic Department of the Institute of Biomedical Technologies - National Research Council (ITB-CNR), located in Segrate (Milan), Italy. GM (PhD), Full Professor and Director at the Computer Science Department of the University of Milano-Bicocca, located in Milan (Italy). PLM (PhD), Principal Investigator at the Proteomic and Metabolomic Department of the Institute of Biomedical Technologies - National Research Council (ITB-CNR), located in Segrate (Milan), Italy.

## Supplementary Material

Additional file 1 Supplementary Figure S1 (PNG file format) — Sample collections and related experimental data selected and used for the study purpose. For each sample five different datasets were used. In addition to the global protein and peptide profiles, m/z precursor ions, specifically detected from the chromatographic steps corresponding to 60, 120 and 400 mM of ammonium chloride concentration, were considered. They cover the central part of the salt gradient elution range (0-700mM) and assure the identification of most of the peptides. Click here for file

Additional file 2 Supplementary Information (PDF file format). Click here for file

Additional file 3 Supplementary Table S1 (PNG file format) — Matrix of high-dimensional proteomic data obtained analyzing sample by means of the MudPIT approach. Rows represent features (e.g., m/z values, peptides or proteins), while columns indicate samples. In each cell it is reported a value corresponding to the parameter associated with feature. In particular, peak area intensity (AUC) was used for *m/z* mass points, Xcorrelation (Xcorr) values for peptides and spectral count (SpC) values for proteins. Click here for file

Additional file 4 Supplementary Figure S2 (PNG file format) — Venn diagram. Venn diagram of differentially expressed proteins identified in collection 1 (A) and collection 2 (B). Evaluation of quantitative level was performed by applying DAve and DCI formulas, G-test and Student’s t-test. In brackets is reported the number of proteins matching with the *features* selected by SVM. Click here for file

Additional file 5 Supplementary Figure S3 (PNG file format) — Rapid Miner workflow. Rapid Miner WF for the Feature selection (a) and model construction/validation (b) phases. Blocks correspond to simple processes in the whole design: each operator receives an input and delivers an output to the forward operator. The function of each block is shortly reported as follow:
 ● **Input Operator** reads data from files.
 ● **Info Gain Weighting Operator** (Fig. a). Each signal is weighted by an information theory criterion (i.e., *info–gain ratio*). The forward phase (Fig. b) employees only signals having weight greater then 0.6;
 ● **Cross Validation Operator** encapsulates a cross validation (*k*–fold) process [37]: the input data set *S* is split up into subsets {*S*_1_*S*_2_*.S*_*k*_}. The inner operators are applied *k* times using at each iteration *i* the set *S*_*i*_as the test set and *S*∖*S*_*i*_as the training set.
 ● **Parameter Optimization Operator** In order for the SVMs to perform as better (and homogeneous) as possible for each datatype, we optimized the learning parameters over the same space of common values. That is, starting from common ranges (for every datatypes the same ranges of values are used) this operator finds the optimal combination (i.e., providing the highest SVM inference performance) of parameter values by using a *cross validation process*. Here, we briefly report the applied common ranges for the selected combinations (some documentation on Rapid Miner can be downloaded at http://rapid-i.com)
 – *SVM.kernel.type* ∈ {ANOVA,DOT,POLYNOMIAL,RADIAL},
 – *SVM.kernel.degree* ∈ {2,…,6},
 – *SVM.C*, *SVM*.* є *∈ {1,1.5,…,8}.
 ● **Training SVM Operator** implements a Support Vector Machine algorithm to deliver an inference model.
 ● **Model Applier Operator** applies the model delivered by the SVM operator.
 ● **Performance Operator** collects the performance evaluation of the classification task and outputs performance measures. Click here for file

Additional file 6 Supplementary Figure S4 (PNG file format) — Principal Component Analysis of peptide, protein and m/z, data of collection 1 and 2. Overview of protein, peptide and mass spectra data matrices performed by Principal Component Analysis (PCA) (15). PCA was applied by RapidMiner software. High-dimensionality of each data matrix was preliminarily reduced by eliminating features identified with an identification frequency (IF) below a certain threshold. In detail, for protein and peptide datasets were retained features with IF>1, while concerning mass spectra datasets were retained features with IF>4. Finally, the principal components that account for most of the variation (PC1-PC2-PC3) in the original multivariate data were plotted in the multidimensional space. Click here for file
