# Supplementary figures and images for: Availability of MudPIT data for classification of biological samples
Source: J Clin Bioinforma. 2013 Jan 14;3:1. doi: 10.1186/2043-9113-3-1 (PMC3563498; doi:10.1186/2043-9113-3-1)

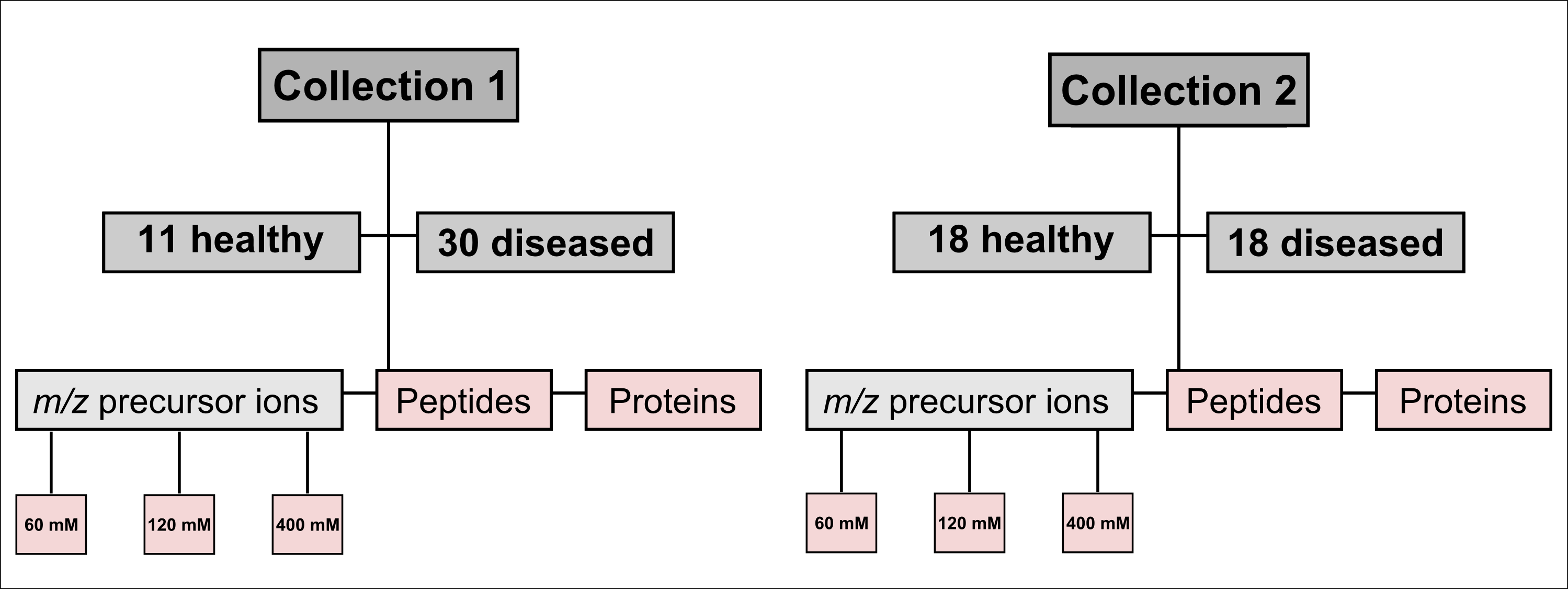

Supplement: Additional file 1 — Supplementary Figure S1 (PNG file format) — Sample collections and related experimental data selected and used for the study purpose. For each sample five different datasets were used. In addition to the global protein and peptide profiles, m/z precursor ions, specifically detected from the chromatographic steps corresponding to 60, 120 and 400 mM of ammonium chloride concentration, were considered. They cover the central part of the salt gradient elution range (0-700mM) and assure the identification of most of the peptides. [file 2043-9113-3-1-S1.png]

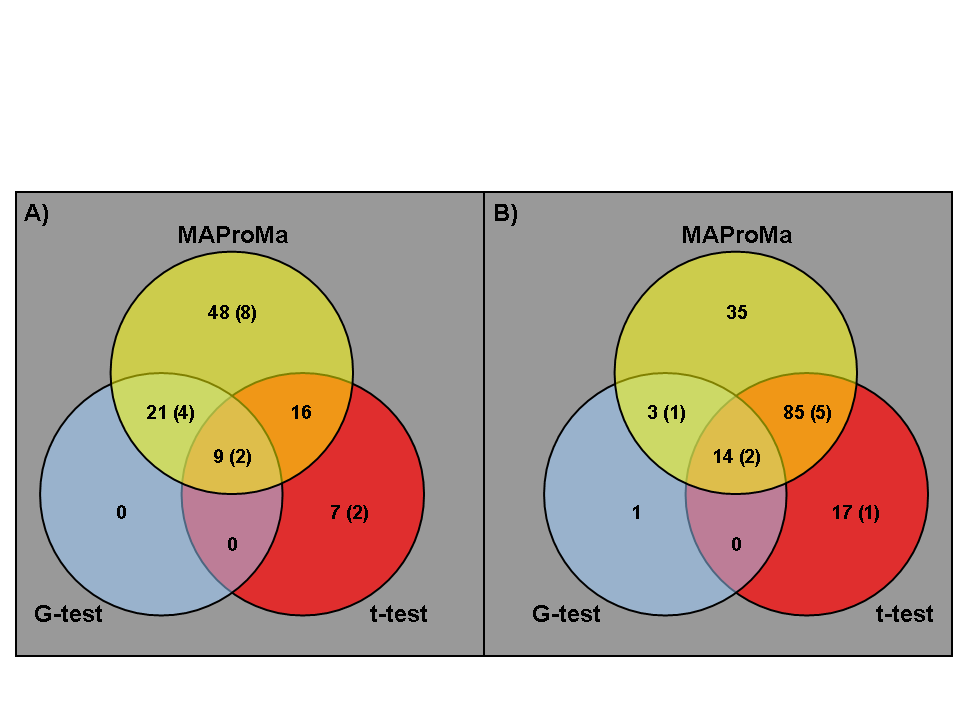

Supplement: Additional file 3 — Supplementary Table S1 (PNG file format) — Matrix of high-dimensional proteomic data obtained analyzing sample by means of the MudPIT approach. Rows represent features (e.g., m/z values, peptides or proteins), while columns indicate samples. In each cell it is reported a value corresponding to the parameter associated with feature. In particular, peak area intensity (AUC) was used for m/z mass points, Xcorrelation (Xcorr) values for peptides and spectral count (SpC) values for proteins. [file 2043-9113-3-1-S3.png]

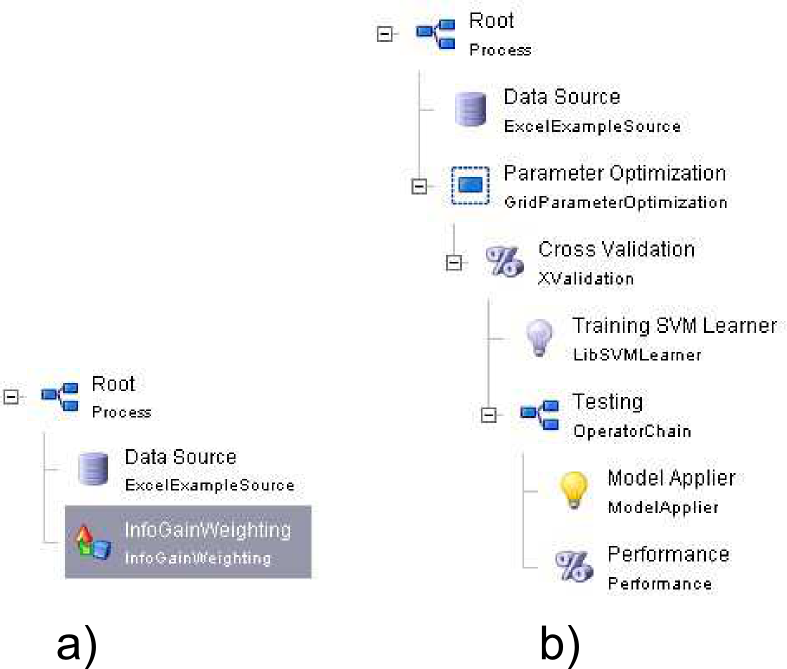

Supplement: Additional file 4 — Supplementary Figure S2 (PNG file format) — Venn diagram. Venn diagram of differentially expressed proteins identified in collection 1 (A) and collection 2 (B). Evaluation of quantitative level was performed by applying DAve and DCI formulas, G-test and Student’s t-test. In brackets is reported the number of proteins matching with the features selected by SVM. [file 2043-9113-3-1-S4.png]

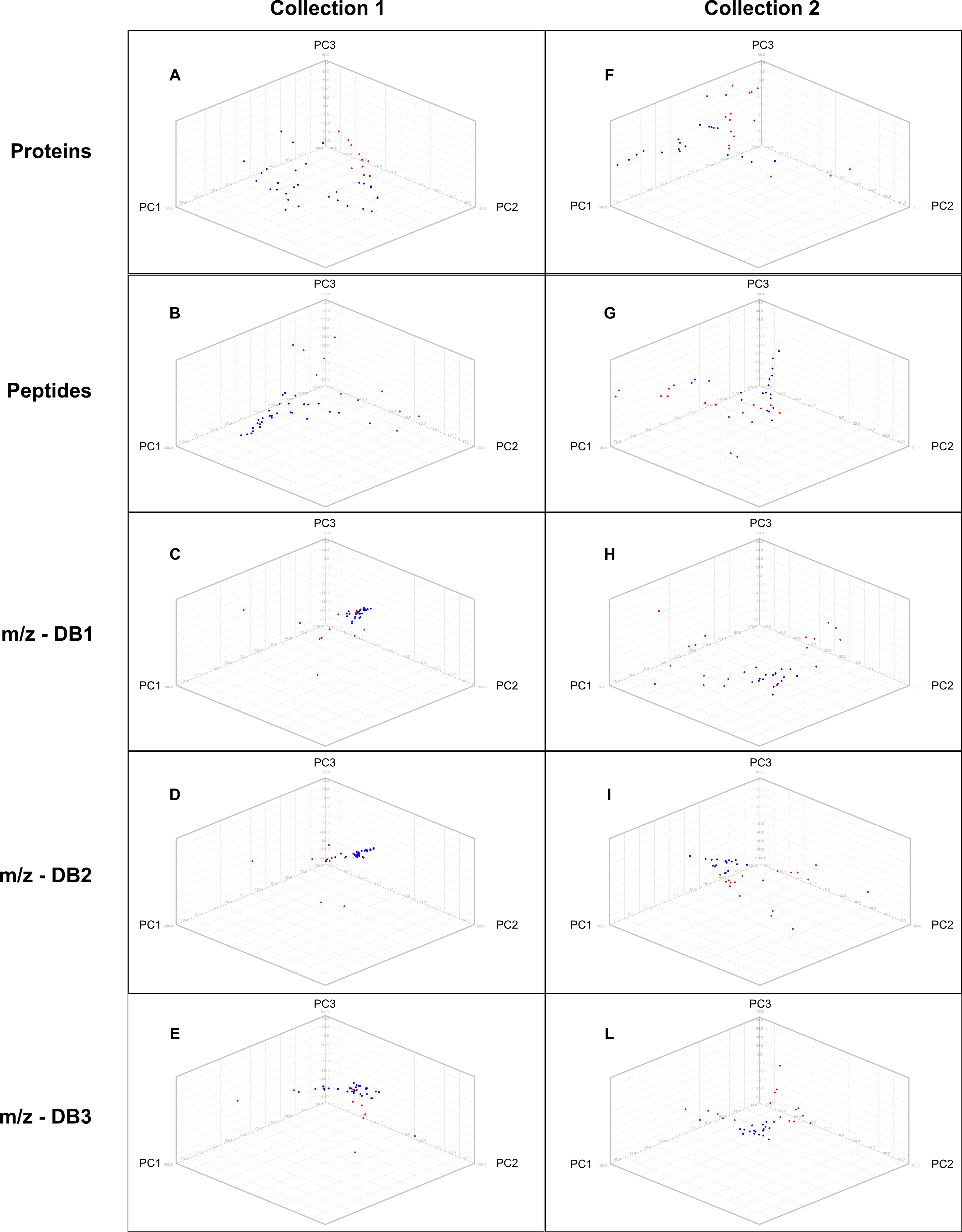

Supplement: Additional file 5 — Supplementary Figure S3 (PNG file format) — Rapid Miner workflow. Rapid Miner WF for the Feature selection (a) and model construction/validation (b) phases. Blocks correspond to simple processes in the whole design: each operator receives an input and delivers an output to the forward operator. The function of each block is shortly reported as follow: ● Input Operator reads data from files. ● Info Gain Weighting Operator (Fig. a). Each signal is weighted by an information theory criterion (i.e., info–gain ratio). The forward phase (Fig. b) employees only signals having weight greater then 0.6; ● Cross Validation Operator encapsulates a cross validation (k–fold) process [37]: the input data set S is split up into subsets {S1S2.Sk}. The inner operators are applied k times using at each iteration i the set Sias the test set and S∖Sias the training set. ● Parameter Optimization Operator In order for the SVMs to perform as better (and homogeneous) as possible for each datatype, we optimized the learning parameters over the same space of common values. That is, starting from common ranges (for every datatypes the same ranges of values are used) this operator finds the optimal combination (i.e., providing the highest SVM inference performance) of parameter values by using a cross validation process. Here, we briefly report the applied common ranges for the selected combinations (some documentation on Rapid Miner can be downloaded at http://rapid-i.com) – SVM.kernel.type ∈ {ANOVA,DOT,POLYNOMIAL,RADIAL}, – SVM.kernel.degree ∈ {2,…,6}, – SVM.C, SVM. є ∈ {1,1.5,…,8}. ● Training SVM Operator implements a Support Vector Machine algorithm to deliver an inference model. ● Model Applier Operator applies the model delivered by the SVM operator. ● Performance Operator collects the performance evaluation of the classification task and outputs performance measures. [file 2043-9113-3-1-S5.png]

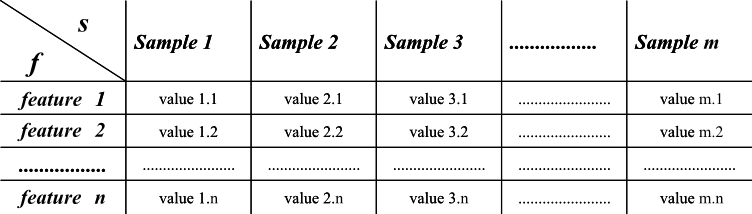

Supplement: Additional file 6 — Supplementary Figure S4 (PNG file format) — Principal Component Analysis of peptide, protein and m/z, data of collection 1 and 2. Overview of protein, peptide and mass spectra data matrices performed by Principal Component Analysis (PCA) (15). PCA was applied by RapidMiner software. High-dimensionality of each data matrix was preliminarily reduced by eliminating features identified with an identification frequency (IF) below a certain threshold. In detail, for protein and peptide datasets were retained features with IF>1, while concerning mass spectra datasets were retained features with IF>4. Finally, the principal components that account for most of the variation (PC1-PC2-PC3) in the original multivariate data were plotted in the multidimensional space. [file 2043-9113-3-1-S6.png]
