# Supplementary material for: Availability of MudPIT data for classification of biological samples
Source: J Clin Bioinforma. 2013 Jan 14;3:1. doi: 10.1186/2043-9113-3-1 (PMC3563498; doi:10.1186/2043-9113-3-1)
Supplement: Additional file 2 — Supplementary Information (PDF file format). [file 2043-9113-3-1-S2.pdf]

Availability of MudPIT data for classification of biological samples

Di Silvestre Dario[a], Zoppis Italo[b], Brambilla Francesca[a], Valeria Bellettato[a], Mauri Giancarlo[b], Mauri Pierluigi[a]

[a] Institute for Biomedical Technologies, ITB-CNR Segrate (Milan), Italy.

[b] Department of Informatics, Systems and Communication University of Milano-Bicocca, Milan, Italy.

Supplementary Information

- **Experimental details of MudPIT analysis**

Trypsin-digested samples were analyzed by MudPIT approach using a ProteomeX-2 system (Thermo Fisher Scientific, San Josè, CA, USA) implemented on a LTQ mass spectrometer. Digested peptide mixtures (5 µl) were first loaded onto a strong cation exchange column (Biobasic-SCX column, 0.32 i.d. x 100 mm, 5 µm, Thermo Electron Corporation, Bellefonte, PA, USA) and eluted stepwise with ammonium chloride salt injections of increasing molarity (0, 20, 40, 60, 80, 120, 200, 400, 600, 700 mM). Fractions were captured in turn onto two peptide traps (Zorbax 300 SB-C18, 0.3 i.d. x 5 mm, 5 µm, Agilent Technologies, Palo Alto, CA) for concentration and desalting prior to final separation by reversed-phase C18 column (Biobasic-18, 0.180 i.d. x 100 mm, 5 µm, Thermo Electron Corporation) with an AcN gradient (eluent A, 0.1% formic acid in water; eluent B, 0.1% formic acid in acetonitrile). Gradient profile was set to 5% eluent B for 5 min, 5% to 40% B in 45 min, 40% to 80% B in 10 min, 80% to 95% B in 5 min, 95% B for 10 min; flow-rate 1 µL/min. Finally, the eluted peptides were electrosprayed directly into a linear ion trap LTQ mass spectrometer equipped with a nano-LC electrospray ionization source (Thermo Finnigan, San Jose, CA, USA). Full mass spectra were acquired in positive mode, and over a 400-2000 *m/z* range, followed by five MS/MS events sequentially generated in a data-dependent manner on the top five most intense ions selected from the full MS spectrum scans using dynamic exclusion for MS/MS analysis.

- **Processing of MS/MS spectra by database search method based on SEQUEST algorithm (SEQUEST defined parameter thresholds)**

Experimental tandem mass spectra (MS/MS) produced by MudPIT runs were matched to in-silico tryptic peptide sequence as previously described (1-2). Briefly, data processing was performed by means of Biowork 3.3.1 software based on SEQUEST algorithm (3). Briefly, matches between spectra were only retained if they had a minimum Xcorr of 2.0 for +1, 2.5 for +2 and 3.5 for +3 charge state, respectively. In addition, maximum value for peptide/protein probability was determined to 10^{-3}, while the minimum value for the SEQUEST-based SCORE was set to 10. Finally, the percentage of the false positives identification was estimated processing the raw mass spectra by means of decoy database (4), and the procedure revealed a false positives rate (FDR) less than 5%. Specifically, FDR was calculated by dividing the number of peptides identified by using the decoy database (reversed) for the number of peptides obtained by target one. Of course, in both cases were used the same SEQUEST defined parameter thresholds**.**

*FDR= (n° peptide decoy database / n° peptide target database) *100*

- **Label-free quantification approaches**

A direct correlation between the SEQUEST-based SCORE values and the relative abundance of the identified proteins has been previously demonstrated (9-10). Based on this findings, protein profiles of healthy and diseased samples were semi-quantitatively compared using a label-free proteomic approach, based on the application of DAve (Differential Average) and DCI (Differential Confidence Index) indices inserted in MAProMa (11). For the study purpose it was applied a value of DAve $\geq$ 0.3 ($\leq$ -0.3), corresponding to a SCORE/SpC ratio $\geq$1.4. As previously demonstrated, it, coupled with a DCI threshold value $\geq$ 300 ($\leq$ -300), allows to identify differentially expressed proteins with a good reliability (2, 9, 12).

To assess the accuracy of our criteria of biomarkers selection and to minimize false identification of differentially expressed proteins, spectral count (SpC) values were further evaluated by using the unpaired Student's t-test and the G-test, as previously described (13). In particular, G-test was calculated according to equation (1) (14):

$$G = 2 f_1 \ln\left(f_1 / \hat{f}_1 \right) + 2 f_2 \ln\left(f_2 / \hat{f}_2 \right) \quad (1)$$

where f_1, f_2 are the normalized SpC for the protein in sample 1 and sample 2, respectively; while $\hat{f}_1$, $\hat{f}_2$ are expected SpC for the protein in sample 1 and sample 2, respectively. All employed statistical analyses were performed with a significance accepted at $P \geq$95%.

- **Rapid Miner**

Rapid Miner (RM) is a software environment for rapid prototyping of machine learning and knowledge discovery processes. It is currently employed for classification (5), clustering (6), and also data integrations tasks (7). In RM, knowledge discovery is modeled by a complex nested chain of objects called operators. The user is supported with graphical interfaces where operators can be dropped as nodes onto the working pane and the data-flow is specified by connecting the operator nodes. In other words, RM workflows represent the conceptual sequence of operational steps employed for different data mining experiment.

Reference

1. Brambilla F, Lavatelli F, Di Silvestre D, Valentini V, Rossi R, Palladini G, Obici L, Verga L, Mauri P, Merlini G: **Reliable typing of systemic amyloidoses through proteomic analysis of subcutaneous adipose tissue**. Blood 2012, 119(8):1844–1847.

2. Simioniuc A, Campan M, Lionetti V, Marinelli M, Aquaro GD, Cavallini C, Valente S, Di Silvestre D, Cantoni S, Bernini F, Simi C, Pardini S,Mauri P, Neglia D, Ventura C, Pasquinelli G and Recchia FA. **Placental stem cells pre-treated with a hyaluronan mixed ester of butyric and retinoic acid to cure infarcted pig hearts: a multimodal study**. Cardiovasc Res, 90(3):546–56, June 2011.

3. Ducret A., Van Oostveen I., Eng J.K., Yates J.R.III and Aebersold R. **High throughput protein characterization by automated reverse-phase chromatography/electrospray tandem mass spectrometry** (1998). Protein Sci., 7:706–719.

4. Wang G, Wu WW, Zhang Z, Masilamani S, Shen RF. **Decoy methods for assessing false positives and false discovery rates in shotgun proteomics** (2009). Anal Chem. Jan 1;81(1):146-59.

5. S. Priya and R. R. Rajalaxmi. Article: **An improved data mining model to predict the occurrence of type-2 diabetes using neural network**. *IJCA Proceedings on International Conference in Recent trends in Computational Methods, Communication and Controls (ICON3C 2012),* ICON3C(3):26–30, 2012.

6. Jupp S, Eales J, Fischer S, Land S, Ramgolam R, Williams A and Stevens R. **Combining rapidminer operators with bioinformatics services - a powerful combination.** In *Rapid-Miner Community Meeting and Conference*, 2011.

7. Zoppis I Gianazza E, Borsani M, Chinello C, Mainini V, Carmen Galbusera C, Carlo Ferrarese C, Gloria Galimberti G, Sandro Sorbi S, Borroni B, Magni F, Antoniotti M, and Mauri G. **Mutual information optimization for mass spectra data alignment**. *IEEE/ACM Trans. Comput. Biol. Bioinformatics,* 9(3):934–939, 2012.

8. T. Mitchell. *Machine Learning*. McGraw-Hill Education, 1997.

9. Mauri P, Scarpa A, Nascimbeni AC, Benazzi L, Parmagnani E, Mafficini A, Della Peruta M, Bassi C, Miyazaki K, Sorio C (2005) **Identification of proteins released by pancreatic cancer cells by multidimensional protein identification technology: a strategy for identification of novel cancer markers.** FASEB J 19: 1125–1127

10. Regonesi ME, Del Favero M, Basilico F, Briani F, Benazzi L, Tortora P, Mauri P, Dehò G (2006) **Analysis of the Escherichia coli RNA degradosome composition by a proteomic approach.** Biochimie 88: 151–161

11. Mauri P and Dehò G. **A proteomic approach to the analysis of rna degradosome composition in escherichia coli.** Methods Enzymol, 447:99–117, 2008.

12. Bergamini G, Di Silvestre D, Mauri P, Cigana C, Bragonzi A, De Palma A, Benazzi L, Döring G, Assael BM, Melotti P, Sorio C: **MudPIT analysis of released proteins in**

Pseudomonas aeruginosa laboratori and clinical strains in relation to pro-inflammatory effects. Integr Biol (Camb) 2012, 4(3):270–279.

13. Zhang B, VerBerkmoes NC, Langston MA, Uberbacher E, Hettich RL and Samatova NF. **Detecting differential and correlated protein expression in label-free shotgun proteomics**. J Proteome Res, 5(11):2909–2918, Nov 2006.

14. Sokal R.R. & Rohlf F.J. (1994) In **Biometry: the principles and practice of statistics in biological research**. Freeman 3rd edition, New York

15. Jackson J.E. A **Users' Guide to Principal Components**. Wiley: New York, 1991.
